# Predictive Simulation of Reaching Moving Targets Using Nonlinear Model Predictive Control

**DOI:** 10.3389/fncom.2016.00143

**Published:** 2017-01-13

**Authors:** Naser Mehrabi, Reza Sharif Razavian, Borna Ghannadi, John McPhee

**Affiliations:** Systems Design Engineering, University of WaterlooWaterloo, ON, Canada

**Keywords:** reaching, NMPC, prediction horizon, motor control

## Abstract

This article investigates the application of optimal feedback control to trajectory planning in voluntary human arm movements. A nonlinear model predictive controller (NMPC) with a finite prediction horizon was used as the optimal feedback controller to predict the hand trajectory planning and execution of planar reaching tasks. The NMPC is completely predictive, and motion tracking or electromyography data are not required to obtain the limb trajectories. To present this concept, a two degree of freedom musculoskeletal planar arm model actuated by three pairs of antagonist muscles was used to simulate the human arm dynamics. This study is based on the assumption that the nervous system minimizes the muscular effort during goal-directed movements. The effects of prediction horizon length on the trajectory, velocity profile, and muscle activities of a reaching task are presented. The NMPC predictions of the hand trajectory to reach fixed and moving targets are in good agreement with the trajectories found by dynamic optimization and those from experiments. However, the hand velocity and muscle activations predicted by NMPC did not agree as well with experiments or with those found from dynamic optimization.

## Introduction

The human central nervous system (CNS), consisting of brain, and spinal cord, is responsible for controlling and maintaining body motions. As first formulated by Bernstein (1967), the CNS simultaneously coordinates the kinematics and kinetics of body motions, despite uncertain/unknown (future) trajectories and the redundancy in muscle actuators. As an example, in a goal-directed planar reaching task, where only the final position of the hand is specified, an infinite solution set of hand trajectories and muscle activation patterns exist to reach the final position. The early observations of reaching and pointing tasks led to the well-known “Minimum-X” models [e.g., minimum-jerk model (Flash and Hogan, 1985; Wada et al., 2001), minimum-torque-change model (Uno et al., 1989), minimum-variance model (Harris and Wolpert, 1998), and minimum-work model (Soechting et al., 1995)] to predict the hand trajectory. These models hypothesize that the CNS coordinates the body movement such that an exertion (X) is minimized. Later, this hypothesis is extended to consider physiologically-motivated exertions such as muscle activation effort (Crowninshield and Brand, 1981; Happee and Van der Helm, 1995; Ackermann and van den Bogert, 2010), metabolic energy expenditure (Anderson and Pandy, 2001; Peasgood et al., 2006), and muscle fatigue (Sharif Razavian and McPhee, 2015).

In computer simulations, the Minimum-X model has been successfully implemented using dynamic optimization (DO) to predict the average human motion for a given task. A common DO approach parameterizes the muscle activation profiles for the period of motion and searches the feasible space to find the profiles that minimize X (Davy and Audu, 1987; Yamaguchi and Zajac, 1990; Neptune and Hull, 1998; Anderson and Pandy, 2001; Kaplan and Heegaard, 2001; Sha and Thomas, 2013; Kistemaker et al., 2014). This approach provides an open-loop (feedforward) command of muscle activations to control the given task. This command can represent the descending command of a well-repeated/well-learned task [e.g., platform diving (Koschorreck and Mombaur, 2011)]. In this model, the CNS only recalls the learned information, and does not intelligently adjust the commands in real-time. However, during conscious voluntary movements, the CNS has to continuously update the motor commands to correct for errors (Todorov, 2004). For example, previous studies (Sarlegna and Pratik, 2015) on pointing and reaching have shown that the CNS constantly updates the hand trajectory based on sensory (feedback) information. This sensory information can be received from vision, proprioception, audition, the vestibular system, and internal models that can predict the motion (Desmurget and Grafton, 2000).

Dynamic optimization implementation of minimum-X models raises an interesting question: does the CNS predict the trajectory at the beginning of the motion? Or does it constantly readjust the trajectory? If the latter is true, how far in advance does the CNS predict the motion, and how does that affect the motion? This article focuses on these questions and provides a computational platform to study the effects of the prediction horizon using optimal feedback control theory. Optimal control methods have been previously used to find a unique solution for motor coordination (Meyer et al., 1988; Loeb et al., 1990; Sporns and Edelman, 1993; Kuo, 1995; Anderson and Pandy, 2001; Todorov and Jordan, 2002b; Liu and Todorov, 2007); however, there are few applications of optimal feedback control to a nonlinear redundantly-actuated musculoskeletal model. The LQR (linear quadratic regulator) and LQG (linear quadratic Gaussian) control methods have been applied to a linear arm model to describe the hand trajectory (Harris and Wolpert, 1998; Todorov and Jordan, 2002a; Liu and Todorov, 2007). Later, to control the nonlinear dynamics of the neuromuscular system, an iterative LQG (iLQG) controller has been developed, in which the nonlinear model is iteratively linearized (Todorov and Li, 2005).

In the present research, a nonlinear model predictive controller (NMPC) with a finite prediction horizon is employed. Predicting infinitely into the future is highly improbable in humans, and a finite prediction horizon allows more realistic simulation. The NMPC allows us to consider the complexity and nonlinearity of the musculoskeletal system without compromising the accuracy and optimality, as occurs using model linearization. It can be formulated to simulate trajectory tracking and goal-oriented tasks with both fixed and moving targets where it only corrects the deviations from the task goal (Todorov and Jordan, 2002a). The NMPC is a simultaneous control method because the optimal trajectory and its required muscular activities are calculated at the same time. To the best of the authors' knowledge, this work is the first use of NMPC for fully predictive simulation of human reaching tasks.

In this research, we are not focused on the source of the sensory information; we assume that the current biomechanical states (posture and velocities) are available to the CNS when necessary. This assumption seems to be valid for healthy individuals, as a wide range of sensory organs is available to sense and transmit information to the CNS. However, a pathological condition might limit the CNS access to this available sensory information. For instance, in a deafferented patient, the sense of position (and therefore the motor skills) is largely lost due to the loss of somatosensory inputs (Bringoux et al., 2016).

This paper is organized as follows. In the Method section, the experimental procedure, the planar arm model, the nonlinear model predictive controller, and forward dynamic simulation framework are provided. Next, in the Results and Discussion section, the use of NMPC as the motor control unit in human reaching tasks is presented and discussed. This study investigates the use of anticipatory planning with continuous error correction by the CNS during reaching tasks. The first goal of this study is to study the effects of varying the prediction horizon on the hand trajectory and muscle activities in a reaching task. Therefore, we ran a number of NMPC simulations with various prediction horizons, as well as a DO simulation to obtain a “gold standard” for comparison. Secondly, the capability of the NMPC as an optimal feedback controller for tracking predefined trajectories has been investigated. This ability is useful when an expected/desired trajectory is available. Lastly, the effectiveness of the proposed NMPC for the simulation of reaching to moving targets is studied. We hypothesize that the anticipatory behavior of the CNS can be modeled by NMPC and verified by comparing the hand trajectory predicted by NMPC to those collected in experiments. Finally, Conclusions and Future Work are presented.

## Methods

### Experiments

**Figure 1:**
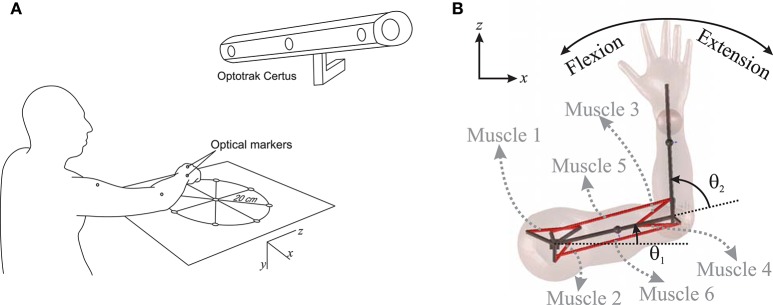
**(A)** Measuring the hand trajectory using NDI Optotrak, **(B)** A schematic of the planar arm model.

To examine the accuracy of the NMPC predictions, a 27 year old male subject was selected to perform reaching tasks. An Optotrak Certus motion capture system (Northern Digital Inc., NDI) was used to measure the arm trajectory at 30 Hz. In these experiments, the subject was seated with the arm elevated at the shoulder level. An active marker attached to the back of the hand (as shown in Figure 1A) has been used to capture center-out hand motion trajectories. The subject was asked to move his hand from an initial central position to one of eight final targets spread evenly on a circle of 20 cm radius at a self-selected convenient speed. The experiment was repeated 10 times for each target with 2 min rest intervals between each set. The subject also performed reaching to moving targets. He was instructed to reach to a target, which was relocated to another position midway through the movement. The subject was instructed to adjust his motion to reach to the moving target. The subject was also given 5 min to rest before performing the reaching moving targets experiment.

During the reaching trails, the electromyography (EMG) activity of seven muscles (anterior/middle/posterior deltoid, long/lateral triceps brachii, biceps brachii, and brachioradialis) were collected at 2000 Hz using a Trigno portable EMG system (Delsys Inc.). The EMGs were band-pass filtered (5–800 Hz cut-off), rectified, low-pass filtered (4 Hz cut-off), and normalized to maximum voluntary contractions (MVCs). We performed a Pearson correlation analysis to investigate the correlation between the EMGs and muscle activity predicted by NMPC and DO simulations. We resampled both the captured EMGs and simulation results with a sampling rate of 100 Hz, and performed the Pearson correlation analysis using the “Corr” command in MATLAB. The experiments have been approved by the Office of Research Ethics at the University of Waterloo and carried out with written informed consent from the subject. The subject gave written informed consent in accordance with the Declaration of Helsinki.

### Planar arm model

In this research, a planar arm model similar to the one developed by Ghannadi et al. (2015) was used. The model consisted of torso, upper arm, and forearm to simulate the hand motion. The torso was fixed and the shoulder and elbow were modeled using revolute joints. Six muscle groups including shoulder and elbow mono-articular flexors/extensors and two bi-articular flexors/extensors were used to actuate the arm as shown in Figure 1B. A modified Hill-type muscle model with muscle excitation-to-activation dynamics was used to simulate the skeletal muscle contraction dynamics (see Appendix A for details). The muscle parameters of the planar arm model (i.e., insertion and origin positions, maximum isometric force, fiber optimal length, slack length, and pennation angle) were tuned to represent the dynamics of the upper extremity in the experimental condition (reaching targets in a horizontal plane elevated at the shoulder level). These parameters were tuned through a series of optimizations so that the planar model provides the same joint torques as a high-fidelity three-dimensional upper extremity model (Ghannadi et al., 2015). Kinematic and dynamic parameters of the arm model and the Hill muscle model parameters used here can be found in Tables 1, 2, respectively.

**Table 1:** **Kinematic and dynamic parameters of the planar arm model**.

**Segment**	**Mass (Kg)**	Inertia [I_yy_] (kg.cm^2^)^*^	**Length (mm)**	**CoM from proximal (mm)**
Upper arm	1.93	141	290	145
Forearm	1.52	188	300	150

* *About the center of mass, the mechanical y axis is assumed to be perpendicular to the plane of movement*.

**Table 2:** **Hill-type muscle model parameters**.

**Muscle**	**ISO force [$F_{0}^{\text{max}}$] (N)**	**Tendon slack length [L_SE_] (mm)**	**Pennation angle [α_*p*_] (deg)**
Shoulder mono-articular flexor (muscle 1)	2525	29.2	21.6
Shoulder mono-articular extensor (muscle 2)	1672	0	19.5
Elbow mono-articular flexor (muscle 3)	1452	18.1	1.4
Elbow mono-articular extensor (muscle 4)	1577	7.2	7.8
Bi-articular flexor (muscle 5)	972	187.6	0
Bi-articular extensor (muscle 6)	798	119.2	12

*The details of how the upper-extremity muscle groups are lumped into these representative muscles can be found in Ghannadi et al. (2015)*.

### Principles of nonlinear model predictive control

**Figure 2:**
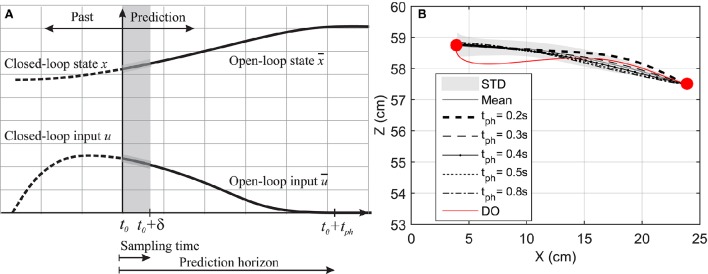
**(A)** Prediction horizon in NMPC. The solid lines shows the optimal muscle activation and state trajectories in the given prediction horizon. **(B)** Hand position trajectory. The hand moves from its original position to the marked position on its left.

The arm motion is controlled by complex commands descending from the CNS, which are the combination of the motion prediction (feedforward control) from an internal representation of the body and environment (or so-called internal model; Desmurget and Grafton, 2000), and the corrective command from the sensory organs to correct any errors due to uncertainty or unknown environment (feedback control). This complexity is captured here by a model-based NMPC with a receding horizon. The NMPC uses a control-oriented model (COM) representing the human's internal model to predict the optimal trajectory, and feedback information to correct the prediction errors. The NMPC predicts the optimal dynamics of the system (*x*, *u*) over a prediction horizon as shown in Figure 2A by minimizing the following cost function:

(1) $$\begin{array}{l} J=\Psi \;\left(t_{0}+t_{ph}\right)+\int_{t_{0}}^{t_{0}+t_{ph}}\psi \;\left(x\left(t\right),u\left(t\right)\right)\;dt \end{array}$$

(2) $$\begin{array}{l} \text{subject to}:\;0<u\left(t\right)<1 \end{array}$$

where Ψ is the cost evaluated at the end of prediction horizon, ψ is the cost evaluated during the prediction horizon, and *t*_*ph*_ is the length of prediction horizon. The state variables at the current time (*t*_*o*_) are obtained from the current sensory information. The input (ū) is an optimal open-loop solution over the prediction horizon. If there are no external disturbances and no model uncertainty in the system, with infinitely long prediction horizon, the open-loop solution can be applied to the system for all time *t* > *t*_*o*_. However, for the finite horizon case and in the presence of noise and uncertainty, the open-loop solution should only be applied until the next sampling time (*t*_0_ + δ). At the new time step, the optimal solution is re-evaluated with the new initial conditions for the receding horizon and iteratively applied to the system. By incorporating the feedback information, the NMPC is converted from a completely open-loop controller to an optimal closed-loop controller. The NMPC can handle constraints on both the states and the inputs. In musculoskeletal models, the muscle activation command must be non-negative and less than one, and constraints on states can be added to avoid unphysiological movements.

### Mathematical formulation of NMPC

In this article, the optimal dynamics over the prediction horizon were calculated using the GPOPS-II optimal control package that utilizes an orthogonal collocation method (Patterson and Rao, 2014). This method is a direct (simultaneous) optimization method in which both states (*x*) and inputs (*u*) are parameterized using a series of connected Legendre polynomials and become part of a Nonlinear Programing (NLP) problem. Here, the arm model described in the previous section was used as the COM of the NMPC and the simulation model for planar reaching and pointing tasks. The dynamic equation of the arm model can be described by:

(3) $$\begin{array}{l} \dot{x}=f\left(x\left(t\right),u\left(t\right)\right),\;x\left(0\right)=x_{0} \end{array}$$

where *x* ϵ ℝ^10 × 1^ are the arm model state variables consisting of shoulder angle and angular velocity plus elbow angle and angular velocity, and muscle activation states, and *x*_0_ is the vector of the initial states. The muscle excitation inputs *u* ϵ ℝ^6 × 1^ represent the ratio of excited motor units to the maximum number of motor units in that muscle.

In this research, for the particular case of a goal-directed reaching task, the terminal cost of the NMPC cost function (Ψ) was removed, and the integral part (ψ) is computed from the summation of two terms: (i) a choice of specific physiological cost function, and (ii) a trajectory tracking error. Therefore, the NMPC cost function shown in (1) is converted to:

(4) $$\begin{array}{l} J=\int_{t_{0}}^{t_{0}+t_{ph}}\left(p{\left(\zeta \left(t\right)-\zeta_{des}\right)}^{2}+q\;G^{M}\left(u\;\left(\text{t}\right)\right)\right)\;dt\; \end{array}$$

where *p* and *q* are cost function weightings, and ζ and ζ_*des*_ are the hand position and its desired final value (in the Cartesian coordinate system), respectively. The simulated hand position ζ varies on the prediction horizon, while the desired final value ζ_*des*_ was kept constant. The hand position is calculated from.

(5) $$\begin{array}{l} \zeta =[L_{1}\cos\text{​}\left(\theta_{1}\right)+L_{2}\cos\text{​}\left(\theta_{1}+\theta_{2}\right),\;L_{1}\sin\text{​}\left(\theta_{1}\right)\\ \;{+L_{2}\sin\text{​}\left(\theta_{1}+\theta_{2}\right)]}^{T} \end{array}$$

where θ_1_ and θ_2_ are shoulder and elbow angles, and *L*_1_ and *L*_2_ are upper arm and forearm lengths, respectively. The physiological cost *G*^*M*^(*u*(*t*)) is defined as:

(6) $$\begin{array}{l} G^{M}=u^{2} \end{array}$$

The term *G*^*M*^ in the cost function represents the neural excitation effort to perform the reaching tasks.

GPOPS-II finds the optimal dynamics of each given horizon by minimizing Equation (4) while satisfying inequality constraints related to muscle excitation (2) and equations of motion (3) using the Sparse Nonlinear Optimization (SNOPT) solver (Gill et al., 2005). An *hp-adaptive mesh refinement method* (Liu et al., 2015) has been used within GPOPS-II to refine the individual interval widths and the polynomial degree to reach a final optimal solution. Then, the first five-percent (e.g., 50 for 1000 ms prediction horizon) of optimal activations are applied to the muscles, and the arm motion is simulated. The new position and orientation of the arm are measured and sent back to the NMPC as initial conditions of the next iteration. In this research, we have assumed that the sensory organs can measure the exact joint angles and angular velocities. Uncertainty can be added to the measurements to account for the noise within the sensory organs, and to simulate the variability in the movement repetition. However, this has not been included in the scope of this work. The optimal muscle activations are shifted and considered as the initial guess of the next iteration.

### Dynamic optimization

In addition to the NMPC simulations, a dynamic optimization (DO) using GPOPS-II was performed to simulate the same task. Unlike the NMPC simulations, which continue until the position tracking error passes a certain threshold, the final simulation time and the final position of the hand are explicitly specified in the DO simulations. The DO cost function is:

(7) $$\begin{array}{l} J=\int_{0}^{t_{f}}\;G^{M}\left(u\;\left(\text{t}\right)\right)\;dt \end{array}$$

(8) $$\begin{array}{l} \text{subject to}:\;0<u\left(t\right)<1,\text{ and }x\left(t_{f}\right)=x_{f} \end{array}$$

where *t*_*f*_ is the final simulation time, and *x*_*f*_ is the state vector corresponding to the target position. The same physiological cost function as in the NMPC simulations (Equation 6) was used in DO to compute the optimal hand position trajectory and muscle activations.

## Results

### Effects of the prediction horizon length

In this section, a goal-directed reaching task is simulated and the effect of prediction horizon length variation on the hand trajectory and muscle activation is studied. Here, the hand is initially at rest and in a natural position ($\theta_{1}=44^{\text{o}}$ and $\theta_{2}=58^{\text{o}}$) and moves toward a target 20 *cm* to the left of its initial position as shown in Figure 2B. This task was simulated using NMPC with 0.2, 0.3, 0.4, 0.5, and 0.8 *s* prediction horizons, and DO with a fixed time duration. In the NMPC simulations, the cost function weightings (*p* = *20* and *q* = *1*) were kept the same. The DO final simulation time was chosen to be 1.5 s, in accordance with the experimental reach duration (1.431 ± 0.176 s in 10 repetitions).

Figure 2B demonstrates the hand trajectories for the aforementioned prediction horizons (*t*_*ph*_). Despite the slight differences between the trajectories, the solutions with 0.4, 0.5, and 0.8 s prediction horizons closely correlate with the experimental results. The Pearson correlation factors between NMPC simulations and the experimental trajectories are 0.9351, 0.9758, 0.9947, 0.9991, 0.9994 respectively for 0.2, 0.3, 0.4, 0.5, 0.8 s. The Pearson factor between the DO and experiment is 0.867.

**Figure 3:**
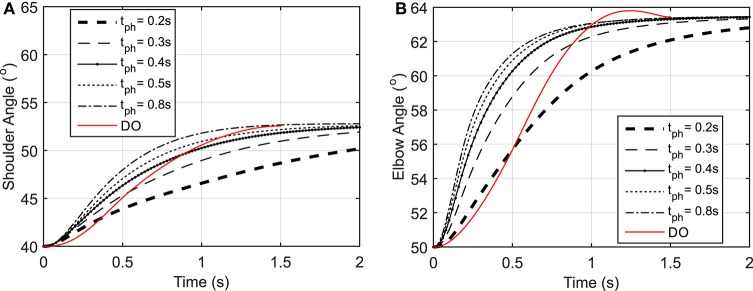
**(A)** Shoulder angle variation during the reaching task, **(B)** Elbow angle variation during the reaching task.

**Figure 4:**
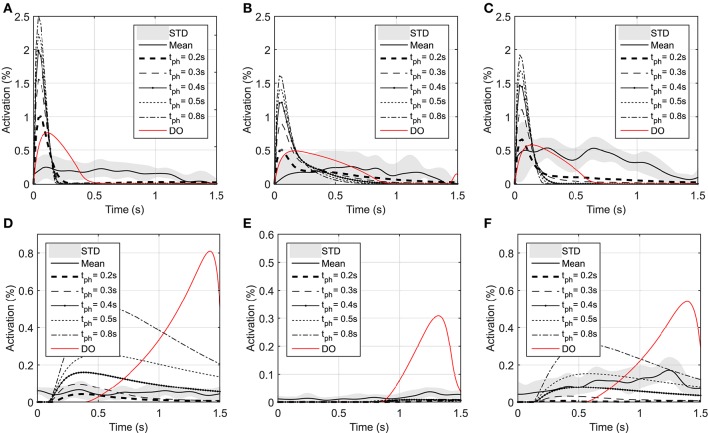
**Optimal activations of flexor and extensor muscles: (A)** Optimal activation of shoulder mono-articular flexor (muscle 1), **(B)** Optimal activation of elbow mono-articular flexor (muscle 3), **(C)** Optimal activation of bi-articular flexor (muscle 5), **(D)** Optimal activation of shoulder mono-articular extensor (muscle 2), **(E)** Optimal activation of elbow mono-articular extensor (muscle 4), **(F)** Optimal activation of bi-articular extensor (muscle 6)

As shown in Figure 3, the elbow and shoulder angle variations with 0.2 *s* prediction horizon are less than those with longer prediction horizons. This signifies the importance of prediction horizon; with short prediction horizons, the controller is more cautious and takes longer to reach a desired position. Simultaneously, this results in smaller muscle activations for shorter horizons since the transient time to reach the final position is longer (see Figure 4). The reaching error at 1.5 s of the simulation is about 23% for 0.2 *s* prediction horizon, and reduces to 0.36% for 0.8 s prediction horizon. As expected for this motion, the shoulder mono-articular flexor (muscle 1), elbow mono-articular flexor (muscle 3) and bi-articular flexor (muscle 5) are activated at the beginning to accelerate the body; then, the antagonistic muscles are activated to reach a full stop at the desired position.

In the DO, similar to NMPC, flexor muscles are active at the beginning of the motion to accelerate the hand toward the target, then the extensor muscles are activated to stop the hand movement (a bang-bang control strategy). Since DO has to stop at the specified final time (1.5 s) the extensor muscle activities are larger than NMPC predictions at the decelerating phase of motion. On the other hand, the flexor muscle activities at the accelerating phase of the simulation are larger for NMPC than DO because the trajectory error at the beginning of the motion is large and exponentially reducing when it gets closer to target.

As shown in Figure 4, the muscle activations predicted by NMPC and DO simulations can capture the general trends of the experimental measurements. It can be observed that as the prediction horizon increases, the NMPC predicts larger muscle activities at the beginning of the motion. A Pearson correlation analysis was performed between the flexor muscle activities predicted by the NMPC and DO simulations and the EMGs from experimental measurements; the correlation coefficients are presented in Table 3. The correlation coefficient for extensor muscles are not reported since the EMG activity of these muscles were minimal in the experiments. As shown in Table 3, the correlation coefficient of flexor muscles reduces when the prediction horizon increases in the NMPC simulations, while the DO predictions correlate better with the experiments.

**Table 3:** **Pearson correlation analysis of flexor muscle activations predicted by NMPC and DO vs. experimental measurements**.

**Muscle**	**Pearson correlation coefficient**					
	**NMPC 0.2 *s***	**NMPC 0.3 *s***	**NMPC 0.4 *s***	**NMPC 0.5 *s***	**NMPC 0.8 *s***	**DO**
Shoulder mono-articular flexor (muscle 1)	0.322	0.300	0.291	0.284	0.276	0.479
Elbow mono-articular flexor (muscle 3)	0.117	−0.051	−0.131	−0.177	−0.214	0.335
Bi-articular flexor (muscle 5)	0.465	0.356	0.297	0.277	0.262	0.565
Average	0.301	0.202	0.153	0.128	0.108	0.422

**Figure 5:**
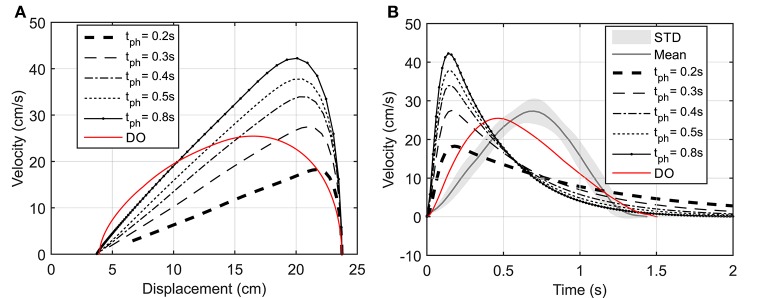
**(A)** Hand speed vs. displacement. In DO simulation, the final time is specified as 1.5 s. **(B)** Hand speed vs. time. The hand moves to a target 20 cm to the left of its initial position.

As suggested by Morasso (1981), subjects tend to move in straight lines with bell-shaped tangential-velocity profiles when reaching for a target. Figure 2B shows that the NMPC with long-enough prediction horizon can realistically predict the hand trajectory, while Figure 5 shows the differences in the velocity profiles of the NMPC and DO predictions. In these simulations, the NMPC tends to accelerate the hand more quickly than the DO; in DO, the optimization knows the final time and can distribute the acceleration over a longer time. On the contrary, in the receding horizon NMPC, the controller can only predict the motion as far as the prediction horizon. This results in the fast acceleration at the beginning the motion due to large tracking errors and slow deceleration at the end of motion due to small tracking errors.

### Reaching targets using a predefined trajectory

**Figure 6:**
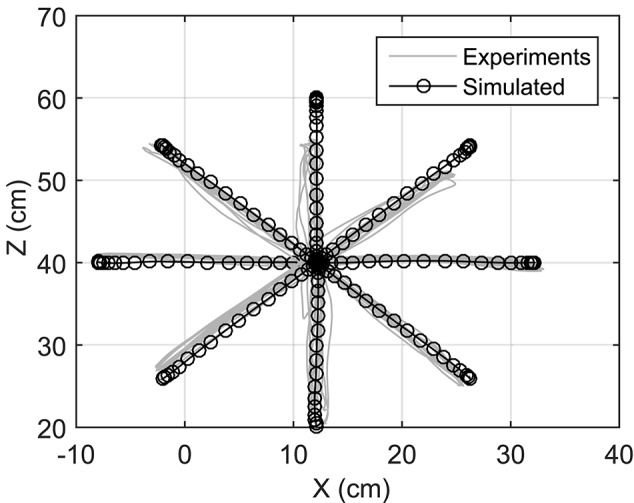
**Optimal trajectories of reaching motions in all directions**.

Reaching to eight different directions was also simulated using NMPC with 0.8 s prediction horizon. The target positions of the hand are located on a circle centered at the initial position of the hand with a radius of 20 cm (see Figure 1). In the NMPC simulations, a smooth 5th-order polynomial with zero initial and final velocities and accelerations is used as the desired straight-line hand trajectory. These trajectories begin at the initial position of the hand and end at the target positions. As shown in Figure 6, the NMPC is able to follow the desired trajectories, which are qualitatively correlated with the measured trajectories in experiments.

### Reaching a moving target

**Figure 7:**
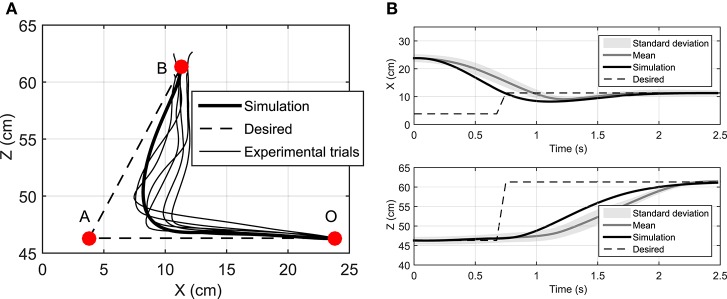
**(A)** The hand trajectory when the target is suddenly moved from **A** to **B**, **(B)** Displacement of hand in X and Z directions when the target is suddenly moved from **A** to **B**.

We have assumed that the CNS plans a trajectory to reach a target and constantly monitors the deviations from this trajectory and the target position. In this section, we study the case where the target position is suddenly relocated. Here, the hand is initially at rest at point O (Figure 7A) and moves toward the target at point A. Then 1 s later, the target position suddenly moves to the point B. This protocol was achieved in the lab by manually moving the target from its initial point A to B when the subject reached half the way to A. In this simulation, the time delay related to the visual cognition of this change [about 150 ms, (Jeannerod, 2006)] has not been considered.

Figure 7 depicts that the NMPC controller can track the location of the target and correct the hand trajectory to reach the new target. As shown in Figure 7A, it seems that when the target moves, the subject over-compensates by moving the hand to the right, while the NMPC finds a trajectory that minimizes both position error and control effort. It is not possible to simulate this scenario with DO; it is one of its disadvantages compared to NMPC, which is able to make online adjustments to the hand trajectory.

## Discussion

In this research, we presented a NMPC to mimic the human motor control system. The results showed that it can successfully replicate certain features of human motor control such as path planning and target tracking. This controller is a fully predictive optimal controller that simultaneously solves the kinematic redundancy and muscle-sharing problem.

In hierarchical models (Menegaldo et al., 2006; Guigon et al., 2007; Mehrabi et al., 2015), the computational burden is reduced by separating the computation into two steps. In the first step, an optimal trajectory is generated based on a kinematic criterion; then, in the second step, the muscle sharing problem is solved based on another criterion (kinetic criterion). The main drawback of hierarchical models is that they follow a preplanned trajectory (output of the first step); therefore the online movement corrections are in favor of trajectory tracking. However, the NMPC controller simultaneously takes into account both kinetic and kinematic exertions to determine an optimal path along with the optimal muscle activations to achieve it. Therefore, it can be argued that this controller is more similar to the human CNS, as it receives proprioceptive information to adjust the predicted trajectory that satisfies the new condition and in favor of the end goal.

Thelen et al. (2003) developed a feedback/feedforward controller (computed muscle control, CMC) that uses inverse dynamics and static optimization to find muscle activities that track a set of desired kinematics. However, such an approach is applicable only if the kinematics is known or if there is a desired kinematics. One advantage of NMPC is its ability to control the motion with and without a prescribed motion, or when the target position moves.

In our study and for the first time, the effect of varying the prediction horizon on the path planning ability in reaching tasks has been investigated. As expected, increasing the prediction horizon improves the tracking performance, but makes the solution computationally more expensive. The prediction horizon length can be adjusted to capture the characteristic of a desired motion. Here, simulation results showed that the resultant hand trajectory with long enough prediction horizons resembles those found from the experiments. However, NMPC accelerates the hand faster and decelerates it slower than the bell-shaped speed trajectories reported by Morasso (1981) and observed in our experiments. This can be due to the fact that the reach time is not specified in the NMPC, or due to the selection of minimum control effort as the physiological cost function. The proposed NMPC is not limited to the suggested cost function; various cost functions can be implemented. For example, Kistemaker et al. (2014) studied the effect of different cost functions on the trajectory of the hand while performing a reaching task using a DO approach.

The Pearson correlation coefficients reported in Table 3 show that muscle activities predicted by an NMPC with a short horizon can better predict (in a temporal sense) the experimental measurements. In contrast, the hand trajectory predictions (in a spatial sense) of an NMPC with larger prediction horizons can more closely replicate those from the experiments as shown in Figure 2B. Finally yet importantly, the NMPC simulations can be used to reproduce the experimental hand trajectories with a moving target as shown in Figure 7. The differences between the experiments and simulations may be due to the subject anticipation of another movement of the target point, while the final target position is known to the controller immediately following the shift. This unique feature of NMPC simulations can advance our theoretical understanding of hand movements and enables the next generation of assistive.

### Online implementation of NMPC

The focus of this paper has been on the proof-of-concept of the NMPC as a possible model for CNS control of human movement. The current implementation of this approach is computationally expensive and is not real-time. For instance, with a prediction horizon of 0.5 s, the NMPC takes 0.45 ± 0.24 s to find the optimal dynamics at each time step and re-plan the movement. These simulations were performed on a computer with an Intel Core™ i7-4790 processor and CPU 3.60 GHZ and RAM 16 GB. However, online NMPC methods such as the Continuation/GMRES method (Ohtsuka, 2004), advanced-step NMPC (Zavala and Biegler, 2009), and explicit MPC (Kouramas et al., 2013) can be used to achieve real-time performance. As an example, Mehrabi et al. (2016) developed a Newton/GMRES NMPC controller to control the functional electrical stimulation of knee extension. This controller by discretising the system dynamics and employing a fast online optimization method (GMRES), significantly reduced the computational time and allowed the real-time implementation of the NMPC. Furthermore, the COM can be further simplified using muscle synergy theory, in which the CNS coordinates human body movements by bundling individual muscles into groups. This allows a low-dimensional control input that significantly reduces of the size of the control problem (Sharif Razavian et al., 2015).

### Conclusions and future work

In this research, the first use of NMPC to simulate human motor control in reaching movements was presented. It was shown that NMPC can replicate certain properties of the human motor control system (i.e., path-planning, prediction, and target tracking), and can be used to realistically simulate reaching movements. Due to its feedback nature, it can correct the tracking errors for static targets or can follow a moving target seamlessly. The NMPC prediction horizon can represent the time horizon for which the CNS minimizes a physiological cost function. It should be noted that the NMPC conclusions from this research are specific to the cost function used here; stronger conclusions can only be made if more diverse cost functions are investigated. Nonetheless, this method opens up new opportunities to study challenging problems such as predictive forward dynamic simulation of biomechanics and biomechatronic systems.

As a possible future research direction, an online NMPC can be used to represent a user/patient in an assistive devices controller to facilitate the shared control between the device and user. This shared control allows the device to perform some tasks independently of the user by sensing information about the environment (Millán et al., 2010). By predicting the motion of the user and adjusting the trajectory online, the NMPC can reduce the cognitive workload imposed on the user, who does not need to consider low-level executions in the presence of external disturbances or obstacles (Tucker et al., 2015). The variability in limb movement is another known characteristic of reaching movements. This characteristic can be incorporated in the NMPC assistive device by accounting for noisy sensory information and sending noisy motor commands to musculotendon units.

## Author contributions

All authors listed, have made substantial, direct and intellectual contribution to the work, and approved it for publication.

### Conflict of interest statement

The authors declare that the research was conducted in the absence of any commercial or financial relationships that could be construed as a potential conflict of interest.

## Figures and Tables

**Figure A1:**
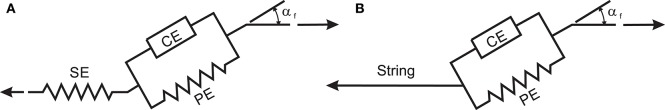
**(A)** Hill muscle model as shown in Thelen et al. (2003), **(B)** Adopted Hill-type muscle model for simulations.

**Figure A2:**
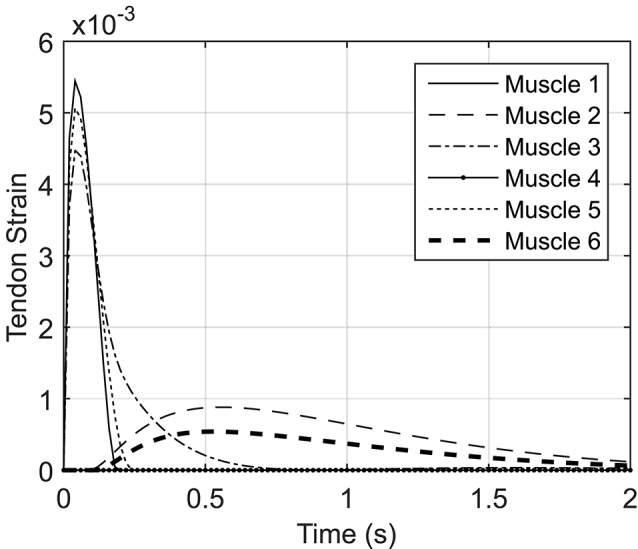
**Simulated tendon strain based on the results for NMPC simulation of reaching with the prediction horizon of 0.5 s**.

## Appendix A

### Hill-type muscle model

In this research, a Hill-type muscle model is used to simulate the muscle contraction dynamics. The Hill muscle model shown in Figure A1A has three elements: contractile element (CE), parallel elastic element (PE), and series elastic element (SE) (Thelen et al., 2003). In this work, we assume that the SE length is constant during the motion; therefore, the SE is replaced with an inextensible string (see Figure A1B). To justify this assumption, we performed an analysis (in Appendix B) that shows that the SE length variation during an NMPC simulation of a straight-line reaching task is negligibly small.

With this assumption, the musculotendon force is simplified to

(A1) $$F_{TM}=F_{0}^{max}\;\left(F_{PE}\left(t,L_{M}\right)+F_{CE}\left(t,a,L_{M},V_{M}\right)\;cos\left(\alpha_{p}\right)\right)$$

where $F_{0}^{max}$, *F*_*CE*_, and *F*_*PE*_ are the maximum isometric force, CE and PE forces, and α_*p*_ is the muscle pennation angle. Here, *L*_*M*_ and *V*_*M*_ represent muscle fiber length and velocity. Muscle fiber length is defined as L_*M*_ = (L_*TM*_ – L_*SE*_) cos(α_*p*_) where L_*TM*_ and *L*_*SE*_ are total length of musculotendon unit and slack length of tendon, respectively. The force generated by *F*_*CE*_ can be separated into force-length and force-velocity relations scaled by the muscle activation command (*a*):

(A2) $$F_{CE}=a(t)F_{CE}^{L}(t,L_{M})F_{CE}^{V}(t,a,L_{M},V_{M})$$

where the force-length ($F_{CE}^{L}$) and force-velocity ($F_{CE}^{V}$) relations are:

(A3) $$F_{CE}^{L}={\text{e}}^{-{\left(\frac{L_{M}}{L_{M}^{opt}}-1\right)}^{2}/\gamma }$$

(A4) $$F_{CE}^{V}=\left\{ \begin{array}{l} \frac{\frac{V_{M}}{V_{M}^{max}L_{M}^{opt}}+AV_{M}^{max}}{-\frac{V_{M}}{V_{M}^{max}L_{M}^{opt}A_{f}+AV_{M}^{max}}}V_{M}<0\\ \frac{\frac{V_{M}B{\bar{F}}_{max}^{len}}{V_{M}^{max}L_{M}^{opt}}+ACV_{M}^{max}}{-\frac{V_{M}B}{V_{M}^{max}L_{M}^{opt}A_{f}+ACV_{M}^{max}}}\;V_{M}>0 \end{array}\right.$$

where γ, A, B, and C are shape factors, $V_{M}^{max}$ is the maximum fiber velocity, $L_{M}^{opt}$ is the optimal length of fiber at which *F*_*CE*_ is a maximum, and ${\bar{F}}_{max}^{len}$ is the maximum normalized muscle force during lengthening. The numerical values of the muscle parameters are taken from (Thelen, 2003).

The Parallel Elastic force of muscle (*F*_*PE*_) is represented by an exponential function:

(A5) $$F_{PE}=\frac{{\text{e}}^{k_{pe}\left(\frac{L_{M}}{L_{M}^{opt}}-1\right)/\epsilon_{0}^{m}}-1}{{\text{e}}^{k_{pe}}-1}$$

where *k*_*pe*_ (= 0.5) is a shape factor and $\epsilon_{0}^{m}$ is passive muscle strain due to maximum isometric force.

In this research, a first-order differential equation based on He et al. (1991) is used to simulate muscle excitation-to-activation dynamics. In this case, muscle activation (*a*) is related to the excitation (*u*) as follows:

(A6) $$\dot{a}=(u-a)(t_{1}u+t_{2})$$

where *u* and *a* are muscle excitation and activation respectively, and *t*_1_ and *t*_2_ are defined as follows:

(A7) $$t_{2}=\frac{1}{\tau_{fall}}\text{ and }t_{1}=\frac{1}{\tau_{rise}}-t_{2}$$

where τ_*fall*_ is the deactivation time constant (= 50 ms), and τ_*rise*_ is the activation time constant (= 15 ms).

## Appendix B

In this section, we have computed the SE strain using the optimal muscle activations from an NMPC simulation. Since the SE element (representing the tendon) is in series with the PE and CE elements of the Hill muscle model, the tendon force is equal to the muscle fiber force, and to that of the musculotendon unit. Therefore, we used the musculotendon force computed in NMPC simulations (with the prediction horizon of 0.8 s) as the tendon force. Then, based on the tendon force-length relation described in Thelen (2003), the tendon strain was calculated using following equation:

(A8) $$F^{T}=F_{0}^{max}\{\begin{array}{ll} \frac{{\bar{F}}_{toe}^{T}}{e^{k_{toe}}-1}\left(e^{k_{toe}\;\epsilon^{T}/\epsilon_{toe}^{T}}-1\right); & \epsilon^{T}\leq \epsilon_{toe}^{T}\\ K_{lin}\left(\epsilon^{T}-\epsilon_{toe}^{T}\right)+{\bar{F}}_{toe}^{T}; & \epsilon^{T}>\epsilon_{toe}^{T} \end{array}$$

where *F*^*T*^ is the tendon force, *k*_*toe*_ (= 3) is an exponential shape factor, *K*_*lin*_ (= $1.712/\epsilon_{0}^{T}$) is a linear scale factor, ${\bar{F}}_{toe}^{T}$ (= 0.33), $\;\epsilon_{0}^{T}$ (= 0.033) is tendon strain due to maximum isometric force, and $\epsilon_{toe}^{T}\left(=0.609\epsilon_{0}^{T}\right)\;$ is the tendon strain above which the tendon exhibits linear behavior. The tendon strain ϵ^*T*^ is defined as $\epsilon^{T}=\frac{L_{T}-L_{SE}}{L_{SE}}$. To find the tendon strain, the first term of the piecewise equation (B.1) was used. If the calculated strain was within the linear region (less than $\epsilon_{toe}^{T}$) the strain value is valid; otherwise the second term of (B.1) was used to calculate the tendon strain.

Figure A2 shows the tendon strain variations during the NMPC simulation of reaching with a 0.8 s prediction horizon. The tendon strains are less than 0.5%; therefore, the SE element of the Hill muscle model can be neglected in the planar arm model.
